# Transgender-Affirming Hormone Therapies, QT Prolongation, and Cardiac Repolarization

**DOI:** 10.1001/jamanetworkopen.2025.24124

**Published:** 2025-07-30

**Authors:** Virginie Grouthier, Marie Matamala, Antoine Tabarin, Amandine Galioot, Thierry Couffinhal, Martino Vaglio, Fabio Badilini, Edi Prifti, Joe-Elie Salem

**Affiliations:** 1 Department of Endocrinology, Diabetes and Nutrition, Centre Hospitalier Universitaire de Bordeaux, Haut-Leveque Hospital, F-33604 Pessac, France; 2 University of Bordeaux, Inserm U1034, Biology of Cardiovascular Diseases, Pessac, France; 3 AMPS LLC, New York, New York.; 4 Unit for Mathematical and Computer Modeling of Complex Systems, Institut de Recherche Pour le Développement, Sorbonne University, Bondy, France.; 5 INSERM, Assistance Publique–Hôpitaux de Paris (AP-HP), Clinical Investigation Center (CIC) 1901, Department of Pharmacology, Pitié-Salpêtrière Hospital, Sorbonne Université, Paris, France

## Abstract

**Importance:**

Transgender women (assigned male at birth) usually take antiandrogens associated with estrogens (or are castrated) to induce feminization, whereas transgender men (assigned female at birth) take testosterone to induce masculinization. However, the cardiovascular outcomes of these gender-affirming hormone therapies (GAHTs) remain poorly studied.

**Objective:**

To examine the association between GAHT intake and cardiac repolarization alterations on electrocardiography in transgender individuals.

**Design, Setting, and Participants:**

In this cohort study, data from a prospective cohort of adult transgender individuals from a single center in France were collected from January 1, 2021, to January 1, 2023. GAHT consisted of injectable testosterone in transgender men and transdermal estradiol with mostly oral cyproterone acetate as antiandrogens in transgender women.

**Main Outcomes and Measures:**

Electrocardiographic features, including QTc, T-wave maximal amplitude (TAmp), and QT peak (QTp; distance between Q onset and T peak), were studied. Circulating sex hormones, including total testosterone, estradiol, progesterone, and gonadotrophins, were assessed concomitantly to electrocardiographic intake.

**Results:**

In the overall cohort of 120 transgender individuals (mean [SD] age, 29.7 [11.9] years; 64 transgender men and 56 transgender women), mean (SD) QTc was similar between 35 transgender women receiving GAHT (406 [20] milliseconds) and 23 transgender men before GAHT (400 [16] milliseconds) but prolonged vs 41 transgender men receiving GAHT (378 [19] milliseconds) (*P* < .001) or 21 transgender women before receiving GAHT (384 [21] milliseconds) (*P* < .001). The start of GAHT in 15 transgender women was associated with increased QTc (mean [SD], 20 [12] milliseconds vs before receiving GAHT; *P* < .001) and decreased QTc in 18 transgender men (mean [SD], −17 [16] milliseconds vs before receiving GAHT; *P* < .001). No participant had a QTc greater than 480 milliseconds or QTc change greater than 60 milliseconds after the start of GAHT in this study. Nonlinear mixed models (eg, integrating age, calcemia, relevant circulating hormones levels, and torsadogenic drug intake) showed that QTc was associated with total testosterone in transgender men (mean [SD] estimate, −1.6 [0.6] ms/ng/mL; *P* = .007) and prolactin (mean [SD], 0.4 [0.1] ms/ng/mL; *P* < .001). In transgender women, QTc was associated with total testosterone (mean [SD] estimate, −3.5 [0.8] ms/ng/mL; *P* < .001). Variation of QTp and TAmp observed after the start of GAHT and associated hormonal alteration were globally associated with those observed with QTc, although in opposite directions for transgender women and transgender men.

**Conclusions and Relevance:**

In this cohort study, testosterone use in transgender men was associated with QTc and QTp shortening and increased TAmp. Androgen deprivation in transgender women was associated with opposite observations. The magnitude of QTc sexual dimorphism seen in cisgender adults was also observed in the transgender population. This work highlights that potential GAHT effects on cardiac repolarization warrant attention in the exponentially increasing transgender population, which is often exposed to coprescribed drugs prolonging QTc and at risk of TdP.

## Key Points

**Question:**

What is the association of gender-affirming hormone therapy (GAHT) with cardiac repolarization, notably QTc (corrected QT interval), a biomarker associated with torsade-de-pointes, in transgender individuals?

**Findings:**

In this cohort study of 120 transgender individuals (64 transgender men treated with testosterone and 56 transgender women treated with antiandrogens), QTc and other T-wave surrogates of cardiac repolarization were measured. GAHT was associated with QTc prolongation in transgender women and QTc shortening in transgender men, with variation in circulating testosterone concentration associated with QTc.

**Meaning:**

In this study, QTc variation after GAHT corresponded in magnitude to restoration of the known QTc sexual dimorphism observed in cisgender adults.

## Teaser

This cohort study of transgender individuals examines the association between gender-affirming hormone therapy intake and cardiac repolarization alterations on electrocardiography.

## Introduction

Transgender medicine has thrived in the past decade, with the transgender population increasing worldwide and representing 0.5% to 3.2% of the general younger adult population.^1,2^ Transgender women (assigned male at birth) usually take antiandrogens associated with estrogens (or are castrated) to induce feminization, whereas transgender men (assigned female at birth) take testosterone to induce masculinization.^1^ However, the cardiovascular impact of these gender-affirming hormone therapies (GAHTs) remains poorly studied, with emerging data showing a potentially increased risk of myocardial infarction, venous thrombosis, or cardiovascular risk factors.^3,4,5,6,7,8^

On electrocardiography (ECG), the QTc (corrected QT interval) represents the duration of cardiac ventricular repolarization corrected for heart rate.^9,10^ An excessive QTc prolongation is associated with an increased risk of torsade de pointes (TdP), a particular form of ventricular arrhythmia that potentially leads to sudden death.^10,11^ Abnormal QTc prolongation and other T-wave alterations are used as surrogates for TdP, such as decreased T-wave maximal amplitude (TAmp) and increased QT peak (QTp; distance between Q onset and T peak), and can be found in some congenital long QT syndrome, but they are mostly drug induced.^10,11,12,13,14^ From puberty to menopause, QTc is 10 to 20 milliseconds longer in women than men, with endogenous testosterone in men and progesterone in women shortening QTc and protecting against TdP, whereas effects of estradiol are inconsistent.^15,16,17,18,19,20,21^ Hypogonadism in men induced by endocrinologic conditions or exogenous hormonal intake (eg, androgen deprivation therapy in prostate cancer) prolongs QTc, decreases TAmp, and subsequently promotes occurrence of TdP.^15,17,22^ In cisgender women, the progestins used in the contraceptive pills with androgenic activity decrease the magnitude of drug-induced QTc prolongation vs progestins with antiandrogenic activity.^16^ In this study, we examine the association between GAHT intake and cardiac repolarization alterations on ECG in transgender individuals, a population with impaired access to care and research programs.^23^

## Methods

### Study Design

**Figure 1.**
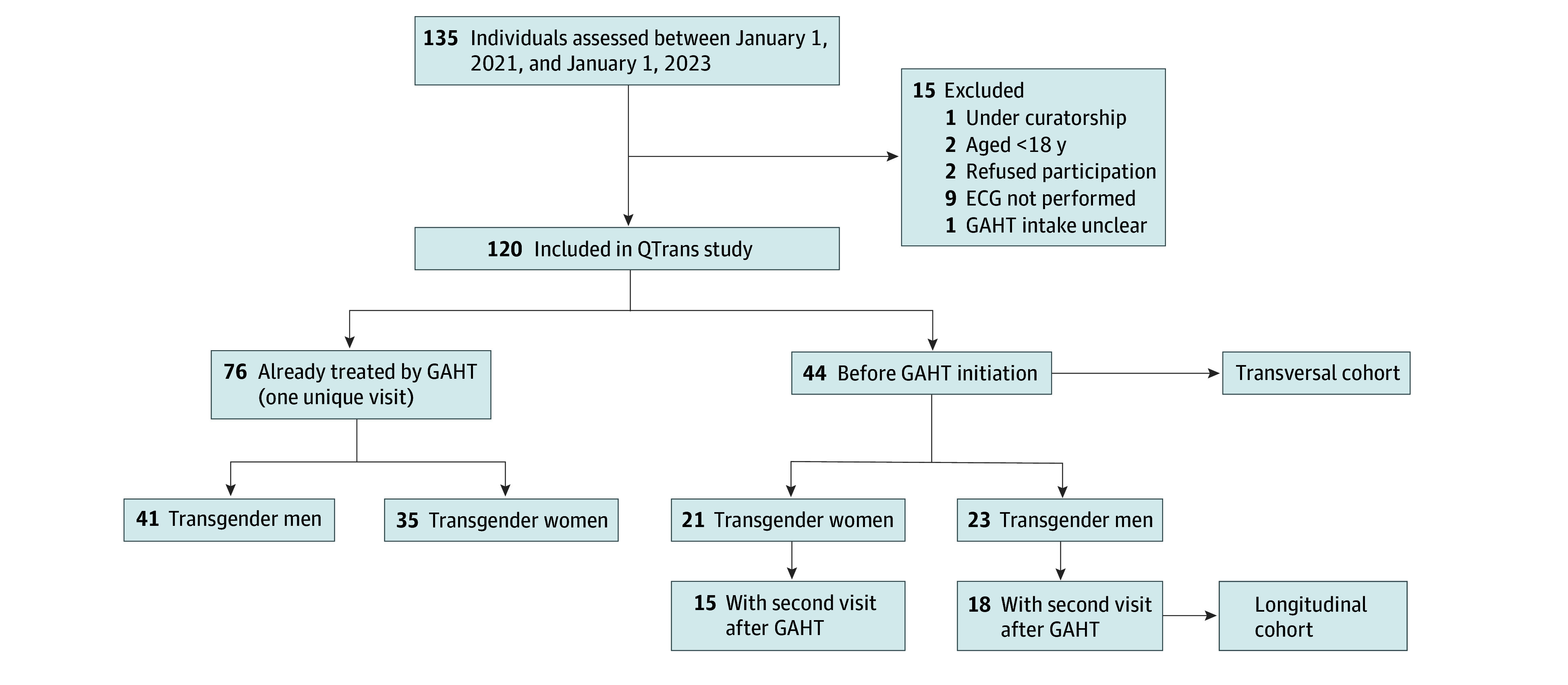
Study Flowchart ECG indicates electrocardiography; GAHT, gender-affirming hormonal therapy; QTrans, TRANS and QT Polarization study.

This prospective single-center cohort study included consecutive adults with gender dysphoria consulting as part of their standard of care at the Department of Endocrinology, Bordeaux University Hospital, Bordeaux, France, for instauration or follow-up of GAHT between January 1, 2021, and January 1, 2023.^24^ Up to 120 individuals were planned to be included until at least 15 transgender men and 15 transgender women had 2 visits with QTc assessed, with the first visit before and the other one after at least 1 month and up to 2 years after the start of GAHT. A total of 135 transgender individuals visited the endocrinology department during the 2-year inclusion period, with 120 patients included (Figure 1). This cohort size (n = 15 per group) has 85% or greater power to detect a difference in QTc of 10 milliseconds or greater after the start of GAHT using a paired *t* test (α = .05; SD of QTc, 12 milliseconds; expected mean QTc, 410 milliseconds and 400 milliseconds in transgender individuals before and after GAHT, respectively; intraindividual correlation = 0.50). Transgender individuals already receiving GAHT at the time of the first visit (inclusion) had only this latter visit. Patients with known congenital long QT syndrome were not eligible for this study. The TdP risk categories for drugs used by participants were checked on CredibledMeds on January 29, 2024.^25^ All patients gave written informed consent to participate, and the study was approved by Bordeaux’s hospital ethics committee. The reporting of the data in this observational cohort study complies with Strengthening the Reporting of Observational Studies in Epidemiology (STROBE) reporting guideline. Sex and gender definitions and terminologies comply with 2022 World Professional Association for Transgender Health guidelines establishing the Standards of Care for the Health of Transgender and Gender Diverse People.^1^

### ECG Acquisition and Analysis

Triplicates of 10 seconds 12-lead digitized ECGs (acquired with BeneHeart R12 devices, Shenzhen Mindray Bio-Medical Electronics Co Ltd) were recorded after few minutes of rest with the participant in the supine position. For each participant, TAmp (in microvolts) and R-R interval (time between 2 R waves), PR interval, QRS complex, QTp, T-peak to T-end (TpTe), and QT interval durations (in milliseconds) were assessed with a semiautomatic approach using the 12-lead overlapped median representative beats generated from digitized ECG using CalECG, version 3.7 (AMPS LLC) (eFigure in Supplement 1) and validated by an expert cardiologist (J.E.S.).^9^ The QT interval was corrected for heart rate using the Fridericia formula (QTc = QT/R-R interval^0.33^). The Fridericia method was chosen in accordance with International Council for Harmonization Guidelines issued from the US Food and Drug Administration in 2005 because this latter correction is more accurate than the Bazett correction in the general population.

### Study Laboratory Analysis

Blood samples were collected on the day of ECG acquisition for the determination of circulating concentrations of sex hormones, potassium, and calcium and assayed in the Bordeaux University Hospital laboratory. Estradiol, progesterone, prolactin, and gonadotrophin (follicle-stimulating hormone and luteinizing hormone) plasma concentrations were assayed by immune chemiluminescence (Architect i2000SR; Roche Diagnostics), and total testosterone levels were measured by liquid chromatography–mass spectrometry.

### Statistical Analysis

Quantitative data are described as median (IQR) or mean (SD) as appropriate. Comparison of the ECG variables between groups in the transversal analysis (4 groups: transgender men or transgender women either naive or receiving GAHT at inclusion) were analyzed with the Kruskal-Wallis test with the Dunn posttest or analysis of variance with the Tukey posttest, as appropriate. Comparison of the quantitative ECG features before and after GAHTs was performed using paired Wilcoxon or *t* test, as appropriate. The univariate correlations among linear variables were assessed with the Spearman or Pearson coefficient, as appropriate, before and after correction for multiple tests (Bonferroni). Nonlinear mixed-effects models (recommended for exposure QT studies)^26^ were used to study within transgender men and transgender women subgroups the association between the main ECG surrogates for TdP (ie, QTc, QTp, and TAmp) using the patient’s identity as the random effect and age, intake of drugs at known risk of TdP,^25^ circulating levels of calcium, total testosterone, and prolactin (only in transgender men for the latter hormone) as fixed effects. A 2-sided *P* ≤ .05 was considered statistically significant, and estimates are presented with SEs or 95% CIs. R software, version 4.4.2 (R Foundation for Statistical Computing) was used to perform the statistical analyses (nlme and lme4 packages).

## Results

### Study Cohort

In the overall TRANS and QT Polarization (QTrans [NCT05865262]) cohort of 120 transgender individuals (mean [SD] age, 29.7 [11.9] years; 64 transgender men and 56 transgender women), 76 were already receiving GAHT at the inclusion visit (41 transgender men and 35 transgender women), and 44 were GAHT naive at inclusion (23 transgender men and 21 transgender women). Of these latter participants, 15 transgender women and 18 transgender men had a follow-up visit after the start of GAHT.

**Figure 2.**
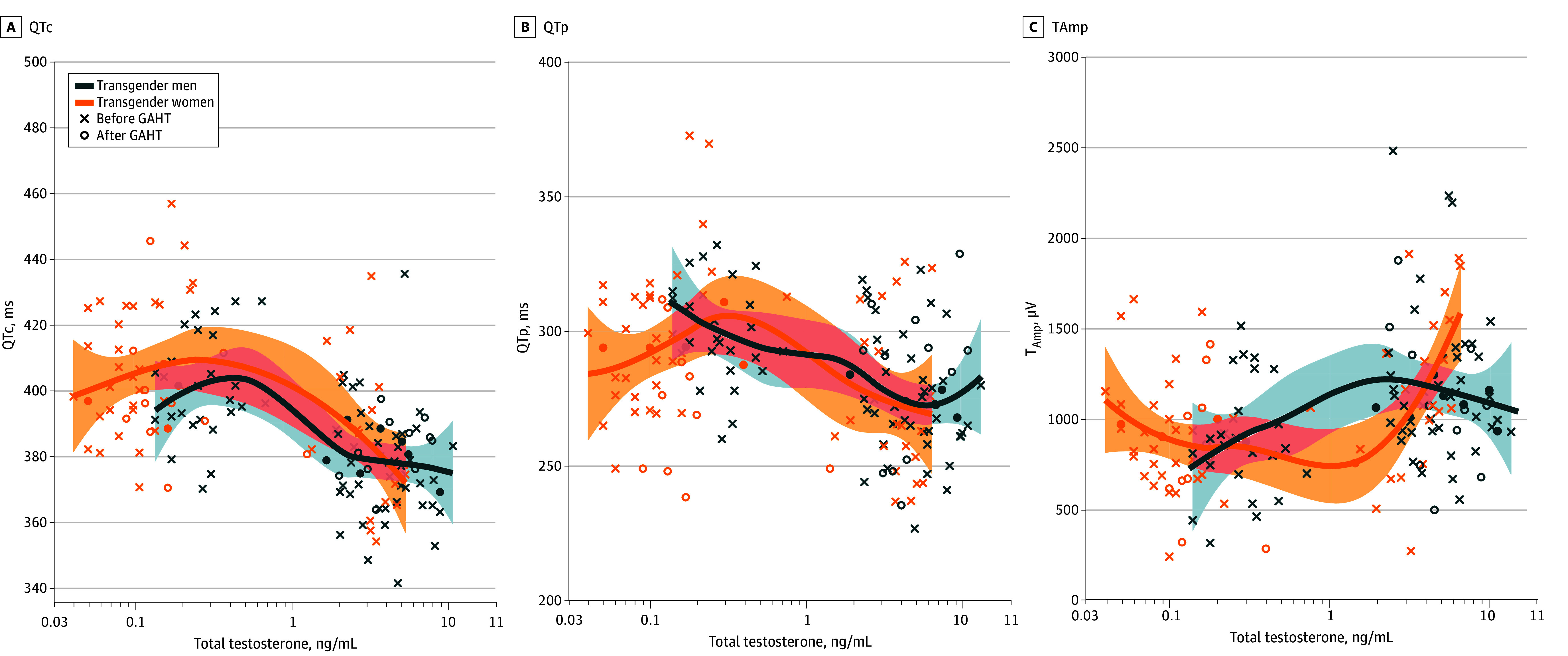
Locally Estimated Scatterplot Smoothing of Corrected QT Interval (QTc), QT Peak Interval (QTp), and T-Wave Maximal Amplitude (TAmp) as a Function of Total Testosterone Levels GAHT indicates gender-affirming hormonal therapy.

The clinical and biological characteristics of the QTrans transversal cohort at inclusion are given in Table 1 (4 subgroups: GAHT-treated or GAHT-naive transgender women and transgender men). The GAHT-treated transgender women were older (median [IQR] age, 37 [27-52] years) than all the other groups (median [IQR], 23 [21-30] years) (*P* < .001). Transgender women were taller and heavier than transgender men. In the overall cohort, the prevalence of hypertension and dyslipidemia was low (3 of 120 dyslipidemia and 2 of 120 hypertension [<3%]), and none had diabetes. Two transgender men and 3 transgender women were taking one drug at known risk of TdP at inclusion (5 of 120 [4%] in the overall cohort).^25^ All 41 GAHT-treated transgender men at inclusion visit were receiving intramuscular testosterone enanthate (median [IQR] dosage, 188 [125-250] ng/mo) started since a median (IQR) of 24 (12-51) months with a last dose received a median (IQR) of 14 (6-21) days before the inclusion visit. The circulating hormonal profile of the 41 GAHT-treated transgender men was within the expected cismen norms (median [IQR] total testosterone, 5.1 [3.0-6.8] ng/mL [to convert to nanomoles per liter, multiply by 0.0347]) (Figure 2).^27^ All the 35 GAHT-treated transgender women (median [IQR], 32 [16-62] months) were receiving 17β-estradiol transdermal gel (median [IQR] dosage, 1.5 [1.0-2.0] mg/d) associated with androgenic deprivation achieved by orchiectomy in 12 transgender women (34%) or chemically induced by oral cyproterone acetate in 19 (54%) (median [IQR] dosage, 50 [25-50] mg/d) and/or oral progesterone in 5 (14%) (median [IQR] dosage, 200 [150-250] mg/d). In the 35 GAHT-treated transgender women, the testosterone levels were almost undetectable (median [IQR], 0.1 [0.1-0.2] ng/mL), and estradiol levels were within the expected age-adjusted range in 34 ciswomen (median [IQR], 68 [42-156] pg/mL [to convert to picomoles per liter, multiply by 3.671]) (Figure 2).^28,29^

**Table 1.** Clinical, Biological, and Electrocardiographic Characteristics at Study Inclusion^a^

Characteristic	GAHT-treated transgender men (n = 41)	GAHT-naive transgender men (n = 23)	GAHT-treated transgender women (n = 35)	GAHT-naive transgender women (n = 21)	*P* value^b^
Clinical					
Age, y	24 (21-30)	20 (19-25)	37 (27-52)	22 (21-33)	<.001^c^^,^^d^^,^^e^^,^^f^
Weight, kg	64 (54-76)	60 (53-70)	77 (69-89)	74 (60-90)	<.001^c^^,^^g^^,^^d^
Height, cm	165 (5)	165 (6)	177 (6)	175 (7)	<.001^c^^,^^g^^,^^f^^,^^h^
BMI	23.0 (20.1-27.1)	22.0 (19.4-25.1)	24.8 (21.9-27.5)	24.9 (19.2-29.4)	.18
SBP, mm Hg	121 (112-132)	112 (109-124)	128 (117-133)	130 (116-134)	.02^f^^,^^h^
DBP, mm Hg	80 (9)	73 (7)	79 (8)	72 (9)	.005^e^^,^^f^
Biological^i^					
Estradiol, pg/mL	34 (26-63)	53 (33-121)	68 (42-156)	23 (19-28)	<.001^c^^,^^g^^,^^e^^,^^h^
Progesterone, ng/mL	0.1 (0.1-0.2)	0.4 (0.3-10.3)	0.1 (0.1-0.2)	0.2 (0.2-0.2)	.001^d^^,^^f^
Testosterone, ng/mL	5.1 (3.0-6.8)	0.3 (0.2-0.4)	0.1 (0.1-0.2)	4.0 (3.0-5.2)	<.001^c^^,^^d^^,^^e^^,^^f^^,^^h^
FSH, mIU/mL	5.1 (3.5-6.6)	5.0 (2.9-6.9)	0.5 (0.1-15.2)	2.6 (1.8-3.8)	.002^c^^,^^g^^,^^f^^,^^h^
LH, mIU/mL	4.8 (2.5-9.4)	4.8 (2.9-9.2)	0.5 (0.5-11.6)	2.1 (1.7-3.5)	.001^c^^,^^g^^,^^f^
Prolactin, ng/mL	13.6 (9.0-20.0)	16.3 (13.3-23.7)	15.0 (10.9-25.1)	9.7 (7.3-12.0)	<.001^g^^,^^e^^,^^h^
Potassium, mEq/L	3.9 (3.8-4.1)	3.9 (3.7-4.1)	3.8 (3.7-4.0)	3.8 (3.4-4.0)	.16
Calcium, mEq/L	2.38 (0.11)	2.33 (0.09)	2.31 (0.1)	2.4 (0.1)	.002^c^^,^^e^
Electrocardiographic^j^					
PR interval, ms	140 (22)	138 (14)	145 (19)	141 (14)	.49
QRS complex, ms	98 (8)	92 (6)	102 (10)	100 (10)	<.001^f^^,^^h^
QTp, ms	272 (262-292)	298 (286-315)	299 (276-314)	265 (251-294)	<.001^c^^,^^d^^,^^e^^,^^h^
TpTe, ms	82 (10)	81 (8)	87 (10)	88 (9)	.03
R-R interval, ms	860 (139)	873 (120)	858 (143)	842 (164)	.91
QTc, ms	378 (19)	400 (16)	406 (20)	384 (21)	<.001^c^^,^^d^^,^^e^^,^^h^
TAmp_,_ μV	1122 (930-1356)	854 (692-1276)	886 (686-1075)	1075 (790-1533)	.003^c^^,^^d^^,^^e^^,^^h^

Abbreviations: BMI, body mass index (calculated as weight in kilograms divided by height in meters squared); DBP, diastolic blood pressure; FSH, follicle-stimulating hormone; GAHT, gender-affirming hormone therapy; LH, luteinizing hormone; TAmp, T-wave maximal amplitude; QTc, corrected QT interval; QTp, QTpeak; SBP, systolic blood pressure; TpTe, T-peak to T-end.

SI conversion factors: To convert estradiol to picomoles per liter, multiply by 3.671; testosterone to nanomoles per liter, multiply by 0.0347; progesterone to nanomoles per liter, multiply by 3.1; FSH and LH to international units per liter, multiply by 1; prolactin to micrograms per liter, multiply by 1; potassium to millimoles per liter, multiply by 1; and calcium to milligrams per deciliter, multiply by 2.0039.

^a^ Data are presented as mean (SD) or median (IQR).

^b^ *P* values are based on multiple comparison posttests.

^c^ Significant for GAHT-treated transgender men vs transgender women.

^d^ Significant for GAHT-treated vs GAHT-naive transgender men.

^e^ Significant for GAHT-treated vs GAHT-naive transgender women.

^f^ Significant for GAHT-treated transgender women vs GAHT-naive transgender men.

^g^ Significant for GAHT-treated transgender men vs GAHT-naive transgender women.

^h^ Significant for GAHT-naive transgender women vs transgender men.

^i^ Biological values are missing due to technical issues for testosterone (n = 1), FSH and LH (n = 2 each), estradiol and potassium (n = 3 each), calcium (n = 8), prolactin (n = 18), and progesterone (n = 27).

^j^ Electrocardiographic values were acquired in triplicate (n = 66), duplicate (n = 18), and single at 10 seconds (n = 36).

The clinical and biological characteristics of the 32 individuals in the longitudinal QTrans cohort are given in Table 2. At inclusion, the 18 transgender men had a median (IQR) age of 21 (19-27) years, and the 15 transgender women had a median (IQR) age of 23 (21-32) years. In both subgroups, weights were associated with a median (IQR) increase of 1 (0-7) kg in transgender men (*P* = .003) and 4 (1-7) kg in transgender women (*P* = .04) between the inclusion and the GAHT follow-up visit, contrasting with unchanged overwhelmingly normal blood pressures at both visits. Only 1 transgender man was taking a drug at known risk of TdP at inclusion, which was stopped, and no participant started drugs at known risk of TdP during the study duration. The 18 transgender men started intramuscular testosterone enanthate (median [IQR] dosage, 125 [125-171] ng/mo) since at least 1 month, with a last dose received a median (IQR) of 8 (4-20) days before the follow-up visit. Median (IQR) testosterone levels increased from almost undetectable (0.3 [0.2-0.4] ng/mL) at inclusion to 4.8 (3.2-7.9) ng/mL while receiving GAHT (*P* < .001). Transgender women started feminizing GAHT-associating 17β-estradiol transdermal gel (median [IQR], 1.5 [1.5-2.3] mg/d) and androgenic deprivation achieved by oral cyproterone acetate in most cases (13 of 15 [87%]; median [IQR] dosage, 50 [25-50] mg/d), except for 1 participant treated with oral progesterone (200 mg/d) and 1 with subcutaneous triptorelin (3 mg/mo). Median (IQR) testosterone levels decreased from 4.3 (3.5-5.3) ng/mL at inclusion to 0.2 (0.1-0.2) ng/mL while receiving GAHT (*P* < .001). Variations observed in other sex hormones, including gonadotrophins and prolactin, were concordant with GAHT intake (Table 2).

**Table 2.** Clinical, Biological, and Electrocardiographic Characteristics of Transgender Men and Transgender Women Before and After GAHT^a^

Characteristic	Transgender men (n = 18)			Transgender women (n = 15)		
	Before GAHT	After GAHT	*P* value	Before GAHT	After GAHT	*P* value
Clinical						
Age, y	21 (19-27)	22 (20-28)	.001	23 (21-32)	25 (22-33)	.007
Weight, kg	61 (55-71)	65 (57-75)	.006	70 (62-87)	74 (63-87)	.04
BMI	22.2 (21.0-25.1)	23.4 (21.2-26.2)	.006	23.9 (18.9-28.9)	24.9 (21.0-27.6)	.02
SBP, mm Hg	116 (111-127)	116 (112-129)	.48	120 (115-134)	129 (122-133)	.63
DBP, mm Hg	74 (6)	77 (8)	.25	72 (8)	74 (9)	.49
Biological^b^						
Estradiol, pg/mL	46 (30-118)	37 (29-86)	.44	23 (20-27)	95 (53-225)	<.001
Progesterone, ng/mL	0.4 (0.3-13.9)	0.3 (0.1-3.4)	.21	0.2 (0.2-0.3)	0.2 (0.1-0.2)	.20
Testosterone, ng/mL	0.3 (0.2-0.4)	4.8 (3.2-7.9)	<.001	4.3 (3.5-5.3)	0.2 (0.1-0.2)	<.001
FSH, mIU/mL	4.4 (2.4-6.4)	3.9 (2.3-5.4)	.27	2.6 (1.8-3.6)	0.1 (0.1-0.2)	.001
LH, mIU/mL	4.1 (2.4-7.6)	3.8 (1.9-5.7)	.10	2 (1.6-2.8)	0.5 (0.5-0.5)	.001
Prolactin, ng/mL	16 (13-20)	13 (10-14)	.03	10 (7-12)	28 (23-37)	<.001
Potassium, mEq/L	3.9 (3.6-4.2)	3.8 (3.6-4.1)	.58	3.8 (3.6-4.0)	3.8 (3.7-3.9)	.84
Calcium, mEq/L	2.32 (0.10)	2.37 (0.07)	.01	2.38 (0.10)	2.32 (0.10)	.03
Electrocardiographic^c^						
PR interval, ms	136 (14)	134 (12)	.53	138 (15)	138 (16)	.88
QRS complex, ms	93 (7)	94 (8)	.56	99 (11)	98 (10)	.48
QTp, ms	299 (284-322)	281 (264-293)	.004	263 (244-267)	288 (249-294)	.03
TpTe	81 (9)	82 (9)	.97	90 (8)	90 (9)	.83
R-R interval, ms	880 (132)	854 (144)	.31	841 (180)	810 (116)	.32
QTc, ms	399 (17)	382 (9)	<.001	379 (18)	398 (17)	<.001
TAmp_,_ μV	824 (656-1277)	1074 (936-1272)	.02	1058 (749-1519)	874 (657-1017)	.02

Abbreviations: BMI, body mass index (calculated as weight in kilograms divided by height in meters squared); DBP, diastolic blood pressure; FSH, follicle-stimulating hormone; GAHT, gender-affirming hormone therapy; LH, luteinizing hormone; TAmp, T-wave maximal amplitude; QTc, corrected QT interval; QTp QTpeak; SBP, systolic blood pressure; TpTe, T-peak to T-end.

SI conversion factors: To convert estradiol to picomoles per liter, multiply by 3.671; testosterone to nanomoles per liter, multiply by 0.0347; progesterone to nanomoles per liter, multiply by 3.1; FSH and LH to international units per liter, multiply by 1; prolactin to micrograms per liter, multiply by 1; potassium to millimoles per liter, multiply by 1; and calcium to millimoles per liter, multiply by 0.5.

^a^ Data are presented as mean (SD) or median (IQR).

^b^ Biological values are missing due to technical issues for testosterone, potassium, and calcium (n = 1); FSH, LH, and prolactin (n = 3 each); and progesterone (n = 11).

^c^ Electrocardiographic values were acquired in triplicate (n = 44), duplicate (n = 8), and single at 10 seconds (n = 14).

### ECG Evaluations

**Figure 3.**
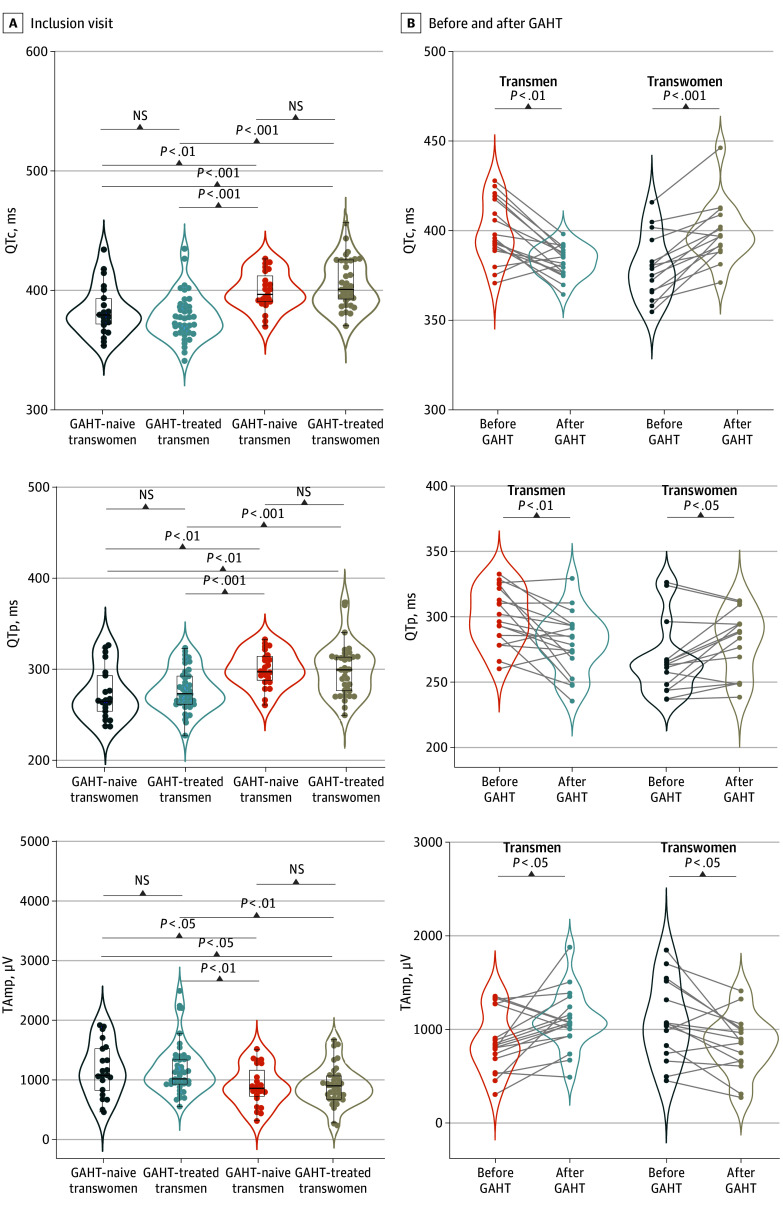
Electrocardiographic Features in Transgender Individuals at the Inclusion Visit and Before and After Gender-Affirming Hormonal Therapy (GAHT) NS indicates not significant; QTc, corrected QT interval for heart rate; TAmp, T-wave with maximal amplitude.

At inclusion, the ECG characteristics of the 120 transgender participants included in the QTrans study are reported in Table 1 and Figure 3A. QTc was similar between the 35 transgender women receiving GAHT (mean [SD], 406 [20] milliseconds) and the 23 transgender men before GAHT (mean [SD], 400 [16] milliseconds) but prolonged vs the 41 transgender men receiving GAHT (mean [SD], 378 [19] milliseconds; *P* < .001) or the 21 transgender women before GAHT (mean [SD], 384 [21] milliseconds; *P* < .001). Concordantly, QTp was shorter in GAHT-treated transgender men (median [IQR], 272 [262-292] milliseconds) and GAHT-naive transgender women (median [IQR], 265 [251-294] milliseconds) vs GAHT-naive transgender men (median [IQR], 297 [286-315] millisecond) and GAHT-treated transgender women (median [IQR], 299 [276-314] milliseconds; *P* < .001). TAmp was higher in GAHT-treated transgender men (median [IQR], 1122 [930-1356] μV) and GAHT-naive transgender women (median [IQR], 1075 [791-1534] μV) vs GAHT-naive transgender men (median [IQR], 854 [692-1276] μV) and GAHT-treated transgender women (median [IQR], 886 [686-1075] μV; *P* = .003). The PR interval, TpTe, and R-R interval did not differ among the subgroups. The QRS complex variations are given in Table 1.

In the longitudinal QTrans cohort (Table 2 and Figure 3B), the start of GAHT in the 18 transgender men was associated with a mean (SD) QTc shortening (−17 [16] milliseconds; *P* < .001) from 399 (17) milliseconds before to 382 (9) milliseconds during virializing GAHT. In the 15 transgender women receiving feminizing GAHT, the mean (SD) QTc was associated with an increase (20 [12] milliseconds; *P* < .001) from 379 (18) milliseconds before to 398 (17) milliseconds during GAHT. Accordingly, QTp was associated with a shortening and TAmp with an increase in transgender men, whereas QTp was associated with a lengthening and TAmp a decrease in transgender women receiving GAHT (Figure 3B). The PR interval, TpTe, QRS complex, and R-R intervals did not change between inclusion and during GAHT visits in either subgroup (Table 2). Overall, in the QTrans study, no participant had a QTc greater than 480 milliseconds at any time point or change in QTc greater than 60 milliseconds observed between values before and after GAHT, and no TdP event was documented.

### Sex Hormones and Cardiac Repolarization

The association between the studied relevant ECG features (ie, QTc, QTp, TpTe) and sex hormone, prolactin, circulating potassium, and calcium levels within the transgender men and transgender women subgroups are given in eTable 1 in Supplement 1 (results of the univariate correlations with *P* value before and significance after correction for multiple tests). Total testosterone level was negatively and significantly associated with QTc in transgender men (ρ = −0.48; *P* < .001) and transgender women (ρ = −0.39; *P* = .01) and QTp in transgender men (ρ = −0.44; *P* < .001). Total testosterone level was marginally positively associated with TAmp in transgender men and transgender women (eTable 1 in Supplement 1). Prolactin levels were associated with QTc transgender men (ρ = 0.51; *P* < .001) and TAmp only in transgender men (ρ = −0.41; *P* < .001). Calcemia was associated with these ECG features in transgender men or transgender women (eTable 1 in Supplement 1). None of the other evaluated sex hormones or potassium levels were associated with these ECG features in transgender men or transgender women (after accounting for multiple tests) (eTable 1 in Supplement 1).

Nonlinear mixed models (eTable 2 in Supplement 1 ) integrating age, calcemia, torsadogenic drug intake (CredibledMeds known risk class),^25^ and total testosterone and prolactin circulating levels, showed in the 53 transgender men that QTc was associated with testosterone (mean [SD] estimate, −1.6 [0.6] ms/ng/mL; 95% CI, −2.8 to −0.5; *P* = .007) and prolactin (mean [SD] estimate, 0.4 [0.1] ms/ng/mL; 95% CI, 0.2-0.6; *P* < .001). In the 54 transgender women, similar nonlinear mixed models showed that QTc was associated with testosterone (mean [SD] estimate, −3.4 [0.8] ms/ng/mL; 95% CI, −5.1 to −1.8; *P* < .001). Nonlinear mixed models also showed that total testosterone was associated with QTp (mean [SD] estimate, −2.0 [0.8] ms/ng/mL; 95% CI, −3.7 to −0.4; *P = *.02) in transgender men and TAmp in transgender women (mean [SD] estimate, 75 [19] μV/ng/mL; 95% CI, 37-112; *P* < .001) (eTable 2 in Supplement 1). The association among QTc, QTp, and TAmp as a function of total testosterone circulating levels in transgender men and in transgender women is displayed in Figure 2.

## Discussion

The QTrans study represents a seminal prospective cohort that included transgender participants before and after the start of GAHT aimed at describing the changes in ECG surrogates used to assess TdP (ie, prolongation of QTc, QTp, and decreased TAmp).^10,11,12,13,14^ We found that feminizing GAHT used in transgender women was associated with a prolongation of QTc and QTp and a decrease in TAmp, whereas masculinizing GAHT used in transgender men was associated with opposite observations. The magnitude of ECG variations, particularly QTc observed among the studied transgender subgroups before and after GAHT, was within 15 to 20 milliseconds and mimicked the magnitude of sexual dimorphism observed in cisgender adults.^30^ The circulating testosterone level, which is an actionable tentative therapeutic target, appeared to be the key hormone associated with ECG alteration observed with GAHT in transgender individuals, including QTc. In transgender men, we identified a novel positive association between prolactin levels and QTc.

### Sexual Dimorphism and Cardiac Electrophysiology

The presence of sexual dimorphism in cardiac electrophysiologic norms and subsequently penetrance of cardiac diseases, such as cardiac channelopathies, is well established.^19,30,31,32^ For instance, Brugada and early repolarization syndromes are more prevalent in men, whereas long QT syndrome affects predominantly women.^30^ Previous studies^19,30,33^ have shown in various physiopathologic situations that QTc is shortened by hyperandrogenism in cisgender women (eg, induced by testosterone or progesterone). Conversely, androgen deprivation in cisgender men, either drug induced (eg, antiandrogens for prostate cancer) or secondary to endocrinologic conditions (eg, hypogonadism), caused QTc prolongation and decreased TAmp, translating into an increased TdP risk and eventually sudden death in cisgender men.^15,22^ With the increased prevalence of transgender, and associated increased use of GAHT, the QTrans study is an important step to formally establish the presence and magnitude of what was a plausible link to be demonstrated between GAHT use and ventricular repolarization alterations.

### Transgender Individuals and Arrhythmias

The current literature on electrophysiologic effects of GAHT is sparse, with the exact type of GAHT, hormonal assessments, and arrhythmias often undefined. In a uncontrolled cohort of 16 555 transgender patients in the US who underwent gender reassignment surgery between 2012 and 2015, in-hospital outcomes were marked by 3.68% cardiac arrhythmia, which was overwhelmingly supraventricular tachycardia but also approximately one-tenth ventricular tachycardia and approximately 0.1% ventricular fibrillation.^34^ These arrhythmias were more common in transgender men than in transgender women, but the potential influence of the type of GAHT and hormonal status was not analyzed.^34^ A Swedish cohort study^35^ of 1779 participants (of whom approximately half had received GAHT) performed between 2006 and 2016 showed an approximately 2- to 4-fold increased rate of conduction disorders reported in transgender men (3.7 per 1000 person-year) and transgender women (4.5 per 1000 person-year) receiving GAHT vs cisgender adults.^35^ In contrast, differences between transgender individuals not receiving GAHT and cisgender individuals were not significant. However, GAHT type and conduction disorder phenotypes were not detailed.^35^ Recently, an ancillary study^36^ of a randomized trial in transgender women tackled the specific issue of QTc variation induced by feminizing GAHT for 6 months (estradiol for all associated with cyproterone or spironolactone) and demonstrated an increase in QTc of approximately 20 milliseconds vs baseline value, which is in line with our findings. Reports exemplifying the actual clinical impact of GAHT on the arrhythmic events and syndromes themselves are rare, with one report^37^ on a transgender man in his 60s developing an aborted cardiac arrest after introduction of testosterone triggered the appearance of a Brugada pattern reversed by normalization of testosterone levels.

### Sex Hormones and Ventricular Repolarization

A major strength of our study specifically designed to assess multiple ECG surrogates tracking ventricular repolarization changes observed before and after the start of GAHT is the deep and standardized ECG and hormonal phenotyping, allowing for a multivariate analysis integrating the major hormonal and drug intake covariates known to influence QTc and TdP risk.^18,25^ Indeed, by using nonlinear mixed models, we can account for within-participant variability, repeated measures, and random effects due to individual differences, which univariate models do not capture. Notably, transgender individuals are particularly subject to psychiatric disorders, leading to coprescription of liable drugs at risk of TdP, especially psychotropics, antidepressants, and opioids.^25,38,39^ Our data further suggest that testosterone is a key hormone associated with human ventricular repolarization alteration in both transgender men and transgender women. This finding is supported by a wealth of preclinical data in several human and animal cardiomyocyte-derived models demonstrating the acute and chronic effects of androgen deprivation (and androgens with opposite effects) to prolong the ventricular action potential and induce ventricular proarythmogenicity.^21,22,30^ Androgen deprivation was repeatedly shown by our group and others^21,30^ to increase the cardiac late sodium current and to decrease the rapid component of the delayed rectifier potassium current, directly favoring or inducing aberrant ventricular depolarization and ultimately TdP.^21,22,30^

The multivariable analysis also identified a positive association between prolactin and QTc only in transgender men (ie, assigned women at birth), which is a novel finding in humans that requires further study to consolidate its causality and mechanism. However, accordingly, in a model of female transgenic long QT2 (ie rapid component of the delayed rectifier potassium current loss) rabbits, Bodi et al^40^ recently identified that prolactin prolonged QTc and cardiomyocytes action potential duration by reducing the slow delayed rectifier potassium current and by increasing Cav1.2 and RyR2 expression and transcription, thereby contributing to an increased ventricular arrhythmic risk.

### Limitations

This study is not without limitations. We acknowledge that the power analysis based on univariate paired *t* test to answer the primary objective led to an overall sample size that limited the statistical power and generalizability of the secondary analyses we performed in this study when integrating multiple covariates in the nonlinear mixed models. This is particularly true for the association studies concerning prolactin and progesterone because there was a substantial rate of missing data for these 2 hormones.

## Conclusions

In this study, feminizing GAHT was associated with QTc prolongation in transgender women, whereas masculinizing GAHT was associated with QTc shortening and TAmp increase in transgender men. These QTc variations associated with GAHT were similar in magnitude to the known QTc sexual dimorphism observed in cisgender adults. Our work highlights that potential GAHT effects on cardiac repolarization warrants attention in the exponentially increasing transgender population, which is often exposed to coprescribed drugs prolonging QTc and at risk of TdP, particularly transgender women.

## Notes

Supplement 1. **eFigure.** Representative Example of Quantitative ECG Measurements Using CalECG3.7 (Overlap Method, AMPS llc) **eTable 1.** Correlation Between Hormones and Main Repolarization ECG Features **eTable 2.** Non-Linear Effects Models Result Studying the Association Between Hormones and Main Repolarization ECG Features

Supplement 2.
Data Sharing Statement
