# Supplementary material for: Transgender-Affirming Hormone Therapies, QT Prolongation, and Cardiac Repolarization
Source: JAMA Netw Open. 2025 Jul 30;8(7):e2524124. doi: 10.1001/jamanetworkopen.2025.24124 (PMC12311718; doi:10.1001/jamanetworkopen.2025.24124)
Supplement: Supplement 1. — eFigure. Representative Example of Quantitative ECG Measurements Using CalECG3.7 (Overlap Method, AMPS llc) eTable 1. Correlation Between Hormones and Main Repolarization ECG Features eTable 2. Non-Linear Effects Models Result Studying the Association Between Hormones and Main Repolarization ECG Features [file jamanetwopen-e2524124-s001.pdf]

Supplemental Online Content

Grouthier V, Matamala M, Tabarin A, et al. Transgender-affirming hormone therapies, QT prolongation, and cardiac repolarization. *JAMA Netw Open*. 2025;8(7):e2524124. doi:10.1001/jamanetworkopen.2025.24124

eFigure. Representative Example of Quantitative ECG Measurements Using CalECG3.7 (Overlap Method, AMPS llc®)

eTable 1. Correlation Between Hormones and Main Repolarization ECG Features

eTable 2. Non-Linear Effects Models Result Studying the Association Between Hormones and Main Repolarization ECG Features

This supplemental material has been provided by the authors to give readers additional information about their work.

© 2025 Grouthier V et al. *JAMA Network Open.*

eFigure. Representative example of quantitative ECG measurements using CalECG3.7 (overlap method, AMPS llc®). The median representative beat is displayed in green (i.e Global Lead in the Results bar)

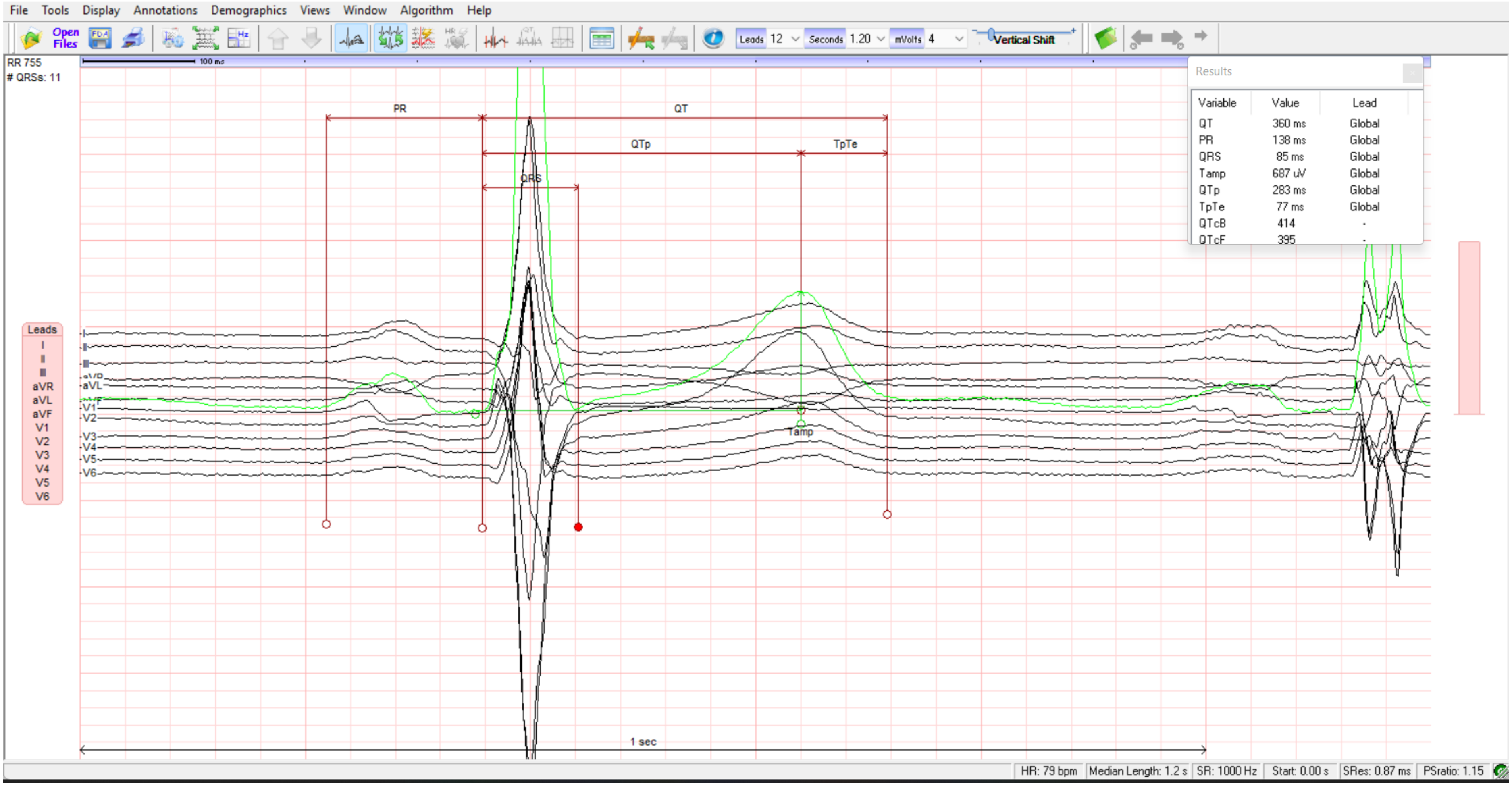

© 2025 Grouthier V et al. *JAMA Network Open.*

eTable-1. Correlation between hormones and main repolarization ECG features

	**Transmen** (women at birth, n=64 individuals, 82 visits)					**Transwomen** (men at birth, n=56 individuals, n=71 visits)				
**Hormones**	**Value**	$N^{evaluation}$	**QTc**<br>*p-value*	**QTp**<br>*p-value*	**TAmp**<br>*p-value*	**Value**	$N^{evaluation}$	**QTc**<br>*p-value*	**QTp**<br>*p-value*	**TAmp**<br>*p-value*
**Estradiol**<br>(pg/mL)	41 [28-75]	80	r=0.21<br>p=0.07	r=0.06<br>p=0.59	r=-0.18<br>p=0.11	55 [23-102]	70	r=0.31<br>p=0.01	r=0.09<br>p=0.47	r=-0.26<br>p=0.03
**Progesterone**<br>(ng/mL)	0.2 [0.1-0.4]	64	r=0.26<br>p=0.04	r=0.26<br>p=0.03	r=-0.20<br>p=0.12	0.1 [0.1-0.2]	61	r=-0.13<br>p=0.33	r=-0.13<br>p=0.33	r=0.26<br>p=0.04
**Testosterone**<br>(ng/mL)	3.3 [0.5-6.2]	82	**r=-0.48**<br>**p<0.0001**	**r=-0.44**<br>**p<0.0001**	r=0.24<br>p=0.03	0.2 [0.1-2.8]	70	**r=-0.39**<br>**p=0.001**	r=-0.19<br>p=0.12	r=0.26<br>p=0.03
**FSH**<br>(UI/L)	4.9 [2.8-6.1]	79	r=0.14<br>p=0.21	r=0.20<br>p=0.07	r=0.07<br>p=0.55	1.1 (0.1-3.8]	69	r=-0.10<br>p=0.43	r=0.05<br>p=0.69	r=0.15<br>p=0.21
**LH**<br>(UI/L)	4.5 [2.4-7.5]	79	r=0.30<br>p=0.01	r=0.20<br>p=0.08	r=-0.15<br>p=0.18	1.1 [0.5-3.1]	69	r=-0.18<br>p=0.14	r=0.04<br>p=0.74	r=0.26<br>p=0.03
**Prolactin**<br>(ng/mL)	14 [11-20]	75	**r=0.51**<br>**p<0.0001**	r=0.19<br>p=0.10	**r=-0.41**<br>**p=0.0002**	13 [10-26]	59	r=0.10<br>p=0.44	r=-0.12<br>p=0.36	r=-0.13<br>p=0.34
**Potassium**<br>(nmol/L)	3.9 |3.7-4.1]	79	r=-0.15<br>p=0.18	r=-0.16<br>p=0.17	r=0.10<br>p=0.36	3.8 [3.7-4.0]	70	r=0.05<br>p=0.69	r=0.16<br>p=0.18	r=0.14<br>p=0.25
**Calcium**<br>(nmol/L)	2.36±0.10	75	r=-0.05<br>p=0.69	r=-0.27<br>p=0.02	**r=0.34**<br>**p=0.003**	2.34±0.11	69	**r=-0.36**<br>**p=0.002**	r=-0.29<br>p=0.01	r=0.29<br>p=0.02

Statistics: p-values without correction for multi-testing (r calculated for Pearson's or Spearman's correlations, as appropriate) are displayed in bold when still significant after correction for multiple testing's (Bonferroni) within transmen or transwomen.

© 2025 Grouthier V et al. *JAMA Network Open.*

eTable-2. Non-linear effects models result studying the association between hormones and main repolarization ECG features

	Transmen (53 subjects, 69 visits)			Transwomen (54 subjects, 68 visits)		
	Estimates ± standard-error, p-values, and (95% confidence interval)			*Estimates ± standard-error, p-values, and (95% confidence interval)*		
	QTc	QTp	T_{Amp}	QTc	QTp	T_{Amp}
**Intercept**	**441±49*** (345, 537)**	**413±73*** (270, 555)**	-2141±1123 (-4344, 60)	**441±49*** (345, 537)**	**361±73*** (217, 505)**	200±1003 (-1766, 2166)
**Age** (years)	-0.4±0.3 (-0.9, 0.1)	0±0.4 (-0.8, 0.7)	12±6 (-0.4, 24)	**0.7±0.2*** (0.4, 1.1)**	**1.1±0.3*** (0.6, 1.6)**	-3.1±3.4 (-9.9, 3.6)
**Drug at TdP known risk**	16±11 (-7, 38)	24±17 (-9, 57)	-306±257 (-810, 197)	13±10 (-7, 33)	-2.5±15 (-32, 27)	2.2±197 (-384, 389)
**Testosterone** (ng/mL)	**-1.6±0.6** (-2.8, -0.5)**	**-2.0±0.8* (-3.7, -0.4)**	9±13 (-17, 34)	**-3.4±0.8*** (-5.1, -1.8)**	-1.3±1.3 (-3.8, 1.2)	**75±19*** (37, 112)**
**Calcium** (nmol/L)	-19±20 (-59, 20)	-51±30 (-110, 7.2)	**1273±462** (367, 2179)**	-26±21 (-67, 14)	-45±31 (-106, 15)	341±424 (-489, 1171)
**Prolactin** (ng/mL)	**0.4±0.1*** (0.2, 0.6)**	0.1±0.2 (-0.2, 0.4)	**-5±2* (-10, -0.7)**	NA	NA	NA

Statistics: * p≤0.05, **p≤0.01, ***p≤0.001

Abbreviations: NA: not applicable because not tested (consistently insignificant in univariate analyses in transwomen, cf. Table-S1)

© 2025 Grouthier V et al. *JAMA Network Open.*
