# Supplementary material for: Transgender-Affirming Hormone Therapies, QT Prolongation, and Cardiac Repolarization
Source: JAMA Netw Open. 2025 Jul 30;8(7):e2524124. doi: 10.1001/jamanetworkopen.2025.24124 (PMC12311718; doi:10.1001/jamanetworkopen.2025.24124)
Supplement: Supplement 2. — Data Sharing Statement [file jamanetwopen-e2524124-s002.pdf]

Data Sharing Statement

Grouthier. Transgender-Affirming Hormone Therapies, QT Prolongation, and Cardiac Repolarization. *JAMA Netw Open*. Published July 30, 2025.
doi:10.1001/jamanetworkopen.2025.24124

Data
Data available: No

Additional Information
Explanation for why data not available: Not GDPR compiant
